# A high-fat, high-saturated fat diet decreases insulin sensitivity without changing intra-abdominal fat in weight-stable overweight and obese adults

**DOI:** 10.1186/1758-5996-7-S1-A234

**Published:** 2015-11-11

**Authors:** Anize Delfino von Frankenberg, Anna Marina, Xiaoling Song, Holly S Callahan, Mario Kratz, Kristina M Utzschneider

**Affiliations:** 1 UFRGS, Porto Alegre, Brazil

## Background

Insulin sensitivity is improved by hypocaloric dietary interventions irrespective of whether they are low or high in fat content, but this effect may be attributed to weight loss itself rather than diet composition.

## Objectives

We sought to determine the effects of dietary fat on insulin sensitivity in weight-stable subjects and whether changes in insulin sensitivity were explained by changes in abdominal fat distribution or very low density lipoprotein (VLDL) fatty acid composition.

## Materials and methods

Overweight/obese adults with normal glucose tolerance consumed a control diet (35% fat/12% saturated fat/47% carbohydrate) for ten days, followed by a four week low fat (LFD, n=10: 20% fat/8% saturated fat/62% carbohydrate) or high fat diet (HFD, n=10: 55% fat/25% saturated fat/27% carbohydrate). All foods were provided and adjusted for weight stability. Insulin sensitivity was measured by labeled hyperinsulinemic-euglycemic clamps, abdominal fat distribution by MRI and fasting VLDL fatty acids by gas chromatography.

## Results

The rate of glucose disposal (Rd) during low- and high-dose insulin, decreased on the HFD but remained unchanged on the LFD (Rd-low: LFD: 0.12±0.11 vs. HFD: -3.67±0.15 mmol/min, mean±SE, p<0.01; Rd-high: LFD: 0.11±0.37 vs. HFD: -0.71±0.26 mmol/min, p=0.08). Hepatic insulin sensitivity did not change. Changes in subcutaneous fat were positively associated with changes in insulin sensitivity on the LFD: r=0.78, p<0.01) with a trend on the HFD (r=0.60, p=0.07), whereas there was no association with intra-abdominal fat. The LFD led to an increase in VLDL stearic, palmitoleic and palmitic acids, while no changes were observed on the HFD. Changes in VLDL 22: 5n6 were strongly associated with changes in insulin sensitivity on both diets (LFD: r=-0.77; p<0.01; HFD: r=-0.71; p=0.02).

## Conclusions

A diet high in fat and saturated fat adversely affects insulin sensitivity and thereby might contribute to the development of type 2 diabetes.

